# A Review on Phytochemistry, Ethnopharmacology, and Antiparasitic Potential of *Mangifera indica* L.

**DOI:** 10.3390/ph18101576

**Published:** 2025-10-18

**Authors:** Diana Mendonça, Yen-Zhi Tan, Yi-Xin Lor, Yi-Jing Ng, Abolghasem Siyadatpadah, Chooi-Ling Lim, Roghayeh Norouzi, Roma Pandey, Wenn-Chyau Lee, Ragini Bodade, Guo-Jie Brandon-Mong, Ryan V. Labana, Tajudeen O. Jimoh, Ajoy Kumar Verma, Tadesse Hailu, Shanmuga S. Sundar, Anjum Sherasiya, Sónia M. R. Oliveira, Ana Paula Girol, Veeranoot Nissapatorn, Maria de Lourdes Pereira

**Affiliations:** 1 CICECO—Aveiro Institute of Materials, University of Aveiro, 3810-193 Aveiro, Portugal; dianapintomendonca@ua.pt (D.M.); sonia.oliveira@ua.pt (S.M.R.O.); 2 School of Health Sciences, IMU University, Kuala Lumpur 57100, Malaysia; 00000043589@student.imu.edu.my (Y.-Z.T.); 00000041786@student.imu.edu.my (Y.-X.L.); 00000042378@student.imu.edu.my (Y.-J.N.); 3 Department of Microbiology, Faculty of Medicine, Infectious Diseases Research Center, Gonabad University of Medical Science, Gonabad 9691833181, Iran; asiyadatpanah@yahoo.com; 4 Division of Applied Biomedical Science and Biotechnology, School of Health Sciences, IMU University, Kuala Lumpur 57100, Malaysia; chooi_linglim@imu.edu.my; 5 Department of Pathobiology, Faculty of Veterinary Medicine, University of Tabriz, Tabriz 516661647, Iran; roghayehnorouzi123@gmail.com; 6 Department of Biotechnology, IILM University, Greater Noida 201310, India; romapandey89@gmail.com; 7 Department of Parasitology, Faculty of Medicine, Universiti Malaya, Kuala Lumpur 50603, Malaysia; wclee@um.edu.my; 8 A*STAR Infectious Diseases Labs (A*IDL), Agency for Science, Technology and Research (A*STAR), Singapore 138648, Singapore; 9 Institute of Advanced Study in Science and Technology, Vigyan Path, Paschim Boragaon, Garchuk, Guwahati 781035, India; ragini.bodade@iasst.gov.in; 10 Biodiversity Research Center, Academia Sinica, Taipei 115, Taiwan; mong432@gmail.com; 11 Center for Health Sciences, Research Institute for Science and Technology, Polytechnic University of the Philippines, Sta. Mesa, Manila 1008, Philippines; rvlabana@pup.edu.ph; 12 Department of Biology, College of Science, Polytechnic University of the Philippines, Sta. Mesa, Manila 1008, Philippines; 13 Department of Microbiology, Icahn School of Medicine at Mount Sinai, New York, NY 10029, USA; jimmmypeace@gmail.com; 14 Department of Microbiology, National Institute of Tuberculosis and Respiratory Diseases, New Delhi 110030, India; ak.verma@nitrd.nic.i; 15 Department of Medical Laboratory Science, College of Medicine and Health Sciences, Bahir Dar University, Bahir Dar 6000, Ethiopia; tadessehailu89@gmail.com; 16 Department of Biotechnology, Aarupadai Veedu Institute of Technology, VMRF (DU), Chennai 603104, India; sundarannauniv85@gmail.com; 17 Gulshan Park, NH-8A, Chandrapur Road, Dist. Morbi (Gujarat), Wankaner 363621, India; sherasiyaanjum@gmail.com; 18 Hunter Medical Research Institute, Lot 1, Kookaburra Circuit, New Lambton Heights, Newcastle, NSW 2305, Australia; 19 Experimental and Clinical Research Center (CEPEC), Padre Albino University Center (UNIFIPA), Catanduva 15809-144, SP, Brazil; anapaula.girol@unifipa.com.br; 20 Futuristic Science Research Center-School of Science, World Union for Herbal Drug Discovery (WUHeDD), and Research Excellence Center for Innovation and Health Products (RECIHP), Walailak University, Nakhon Si Thammarat 80160, Thailand; 21 Department of Medical Sciences, University of Aveiro, 3810-193 Aveiro, Portugal

**Keywords:** bioactive compounds, ethnopharmacology, mango, neglected tropical diseases, parasitic diseases

## Abstract

Parasitic infections remain a major global health challenge, particularly in resource-limited settings where they are closely tied to poverty and inadequate sanitation. The increasing emergence of drug resistance and the limited accessibility of current therapies highlight the urgent need for novel, safe, and affordable alternatives. *Mangifera indica* L. (mango), a widely cultivated fruit tree deeply rooted in traditional medicine, has long been used to treat conditions symptomatic of parasitic diseases, including fever, diarrhea, and dysentery. Phytochemical investigations have revealed a rich spectrum of bioactive compounds, notably mangiferin, phenolic compounds and terpenoids, which exhibit antimicrobial, antioxidant, and immunomodulatory activities. This review critically synthesizes evidence on the antiparasitic potential of *M. indica* against protozoa, such as Plasmodium, Leishmania, Trypanosoma, *Toxoplasma gondii*, *Entamoeba histolytica*, and free-living amoebae, as well as helminths. Strongest evidence exists for malaria and helminth infections, where both crude extracts and isolated compounds demonstrated significant activity in vitro and in vivo. Encouraging but limited findings are available for leishmaniasis and trypanosomiasis, while data on toxoplasmosis and amoebiasis remain largely speculative. Variations in efficacy across studies are influenced by plant parts and extraction methods, with ethanolic extracts and mangiferin often showing superior results. Despite promising findings, mechanistic studies, standardized methodologies, toxicological evaluations, and clinical trials are scarce. Future research should focus on elucidating molecular mechanisms, exploring synergistic interactions with existing drugs, and leveraging advanced delivery systems to enhance bioavailability.

## 1. Introduction

Parasitic diseases continue to exert a significant global health burden. Nearly one-fourth of the world population is affected [1], particularly in low- and middle-income countries, where they are strongly associated with poverty, poor sanitation, and inadequate healthcare access. According to the World Health Organization, malaria alone accounted for approximately 263 million cases and 597,000 deaths worldwide in 2023, with young children and pregnant women representing the most vulnerable groups [2]. Leishmaniasis affects an estimated 600,000–1 million people annually in its cutaneous form, though the less common visceral type poses even greater health risks [3]. Trypanosomiasis, encompassing both African sleeping sickness and American Chagas disease, continues to cause considerable morbidity and mortality among affected populations [4]. Toxoplasmosis affects nearly one-third of the global population, with severe consequences in immunocompromised patients and during pregnancy [5]. More recently, *Acanthamoeba* infections—manifesting as keratitis or granulomatous amoebic encephalitis—have emerged as a growing public health concern, particularly among contact lens wearers and immunocompromised individuals [6].

Beyond protozoan infections, helminthiases remain widespread. Soil-transmitted helminths, including *Ascaris lumbricoides*, *Trichuris trichiura*, and hookworms, collectively infect more than 1.5 billion people worldwide [7]. Schistosomiasis, caused by *Schistosoma* spp., affects over 250 million people, primarily in sub-Saharan Africa, and is considered among the most severe neglected tropical diseases (NTDs) [8].

Although chemotherapeutic agents exist for many of these infections, their efficacy is increasingly undermined by drug resistance. Resistance to artemisinin and its derivatives in *Plasmodium falciparum*, [9,10,11,12], resistance to pentavalent antimonials in Leishmania [13], and benzimidazole resistance in helminths [14] highlight the fragility of current treatment options. Reliance on a single drug, praziquantel, for schistosomiasis further compounds concerns over future treatment efficacy [15]. Moreover, several infections, including acanthamoebiasis, still lack safe or effective therapies [16]. High treatment costs, poor accessibility in endemic areas, and adverse drug reactions underscore the urgent need for sustainable alternatives.

Medicinal plants offer a promising reservoir of bioactive compounds for antiparasitic drug discovery, as exemplified by the discovery of artemisinin from the plant *Artemisia annua* [17]. Across cultures, ethnomedicinal traditions have long relied on botanical preparations to manage parasitic diseases, providing a foundation for modern pharmacological exploration [18,19]. Within this context, *Mangifera indica* L. (Anacardiaceae), commonly known as the mango tree, emerges as a plant of particular interest. Beyond its value as a widely cultivated fruit, *M. indica* has been used in traditional medicine across Africa, Asia, and Latin America, where it is endemic, to treat fever, diarrhea, and dysentery, many of which are linked to parasitic diseases [20,21].

Phytochemical studies have identified a diverse range of bioactive compounds in *M. indica*, including xanthones (notably mangiferin), phenolic acids (gallic, caffeic, ellagic, and chlorogenic acids), flavonoids (quercetin, catechins, kaempferol, and rutin), tannins, triterpenes, and sterols [22,23,24]. These constituents exhibit a broad spectrum of pharmacological activities—antimicrobial, anti-inflammatory, immunomodulatory, and antioxidant—which have been extensively documented [25,26,27]. However, their antiparasitic potential remains comparatively underexplored.

The objective of this review is to provide a focused and critical synthesis of the evidence on the antiparasitic activity of *M. indica*. Specifically, it examines its potential against protozoan diseases (malaria, leishmaniasis, trypanosomiasis, toxoplasmosis, amoebiasis, and acanthamoebiasis) as well as helminthiases. By integrating ethnopharmacological knowledge with experimental findings, this review highlights the therapeutic relevance of *M. indica*, explores potential mechanisms of action, and identifies key research gaps that must be addressed to translate traditional applications into evidence-based antiparasitic therapies.

## 2. Classification and Morphological Description of *M. indica*

**Figure 1:**
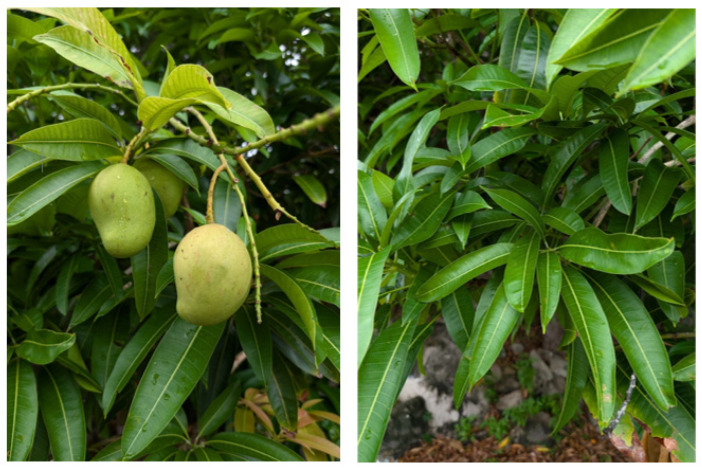
*Mangifera indica* L. observed in Australia by Rebecca (licensed under http://creativecommons.org/licenses/by-nc/4.0/). Reproduced from https://www.gbif.org/, 2025 (accessed on 10 September 2025).

*M. indica* is a long-branched evergreen tree widely recognized for its fleshy, sweet, and aromatic fruit (Figure 1). Scientifically, it belongs to the kingdom Plantae, phylum Tracheophyta, class Magnoliopsida, order Sapindales, family Anacardiaceae, genus *Mangifera*, and species *Mangifera indica*. Native to South and Southeast Asia, mango is now cultivated throughout the tropics and subtropics, thriving in hot and humid climates [28]. The genus *Mangifera* comprises 69 species, including *Mangifera acutigemma* from northeastern India, particularly Sikkim; *Mangifera altissima*, distributed across the Philippines, Indonesia, Malaysia, Papua New Guinea, and the Solomon Islands; and *Mangifera caloneura*, a fruit-bearing tree native to mainland Southeast Asia [29].

*M. indica* is a large tree with a broad canopy, often reaching up to 40 m in height. The bark is thick, brownish-grey, and cracked on the surface. Leaves vary considerably in shape—ranging from lanceolate and oblong-elliptic to oval or slender forms—and measure 15–45 cm in length with petioles 1–10 cm long [30,31]. Young leaves are typically reddish or orange-pink due to anthocyanin pigments, which protect them from herbivores and sunlight, later turning green as chlorophyll accumulates [32,33]. The flowers, borne in large branched panicles at the ends of branches, are small, white to yellowish, and can be male or bisexual, with the ratio varying by cultivar and environment. Fruits, which are drupes, display wide variation in size, shape, skin color, and pulp characteristics across more than 1000 known cultivars. Ripe fruit skin may be green, yellow, orange, or red, while the flesh is typically orange, fibrous or buttery, and sweet. Seeds are oblong or ovoid, enclosed in a tough, fibrous endocarp or “stone” [34].

Mango fruits are considered nutritionally rich, providing essential amino acids such as valine, methionine, cysteine, and isoleucine; vitamins A and C; minerals including calcium, magnesium, zinc, and iron; carotenoids such as β-carotene; sugars like maltose, glucose, and fructose; and dietary fiber [35]. Beyond their dietary value, different parts of the plant—stem, leaves, bark, flowers, and roots—are widely used in traditional medicine across cultures. Extracts from the leaves, bark, seeds, and fruit have demonstrated antimicrobial, antifungal, and antiparasitic activities, as well as analgesic, gastroprotective, hepatoprotective, antidiabetic, antihyperlipidemic, and anticancer properties in various experimental models [36,37,38,39,40,41,42].

Overall, *Mangifera indica* is not only a globally important fruit crop but also a plant of significant medicinal value, with diverse applications rooted in traditional practices and increasingly validated by modern pharmacological studies.

## 3. Phytochemical Constituents of *M. indica* and Their Pharmacological Properties

The therapeutic properties of *M. indica* are largely attributed to its diverse phytochemical composition. Phytochemical characterization has revealed the presence of xanthones, phenolic acids, flavonoids, tannins, triterpenes, and sterols as the most common and relevant groups of compounds (Table 1) [22,23,24].

Xanthones, particularly mangiferin, represent one of the plant’s hallmark constituents. This C-glucosylxanthone is abundant in leaves and bark, and it has been widely recognized for its potent antioxidant properties, alongside its ability to modulate inflammatory pathways and influence immune responses [43]. Such characteristics make mangiferin a central candidate in explaining the broader pharmacological relevance of *M. indica*, particularly in the treatment of parasitic diseases [44].

Phenolic acids, including gallic, caffeic, chlorogenic, and ellagic acids, are distributed across the plant but are usually more abundant in the peel [53]. These compounds are known for their capacity to scavenge free radicals [45], regulate oxidative stress [46] and inhibit microbial growth through mechanisms such as enzyme inhibition [47].

Flavonoids are another group of compounds, with quercetin, catechins, kaempferol, and rutin being the most prominent representatives [23,48]. These molecules are often associated with anti-inflammatory and antioxidant effects [49], as well as the ability to stabilize cell membranes and regulate signaling cascades [50].

Tannins, both in condensed and hydrolysable forms, are present in several parts of the plant, such as leaf, fruit, kernel and stem bark [27]. Their astringent nature enables them to interact strongly with proteins and polysaccharides, resulting in antimicrobial, anti-diarrheal, and tissue-protective properties [51,52].

Finally, *M. indica* also yields a spectrum of triterpenes and sterols, including lupeol, β-sitosterol, and cycloartane derivatives. These compounds are recognized for their anti-inflammatory and immunomodulatory effects, which complement the actions of the plant’s polyphenols [20].

Taken together, the phytochemical composition of *M. indica* illustrates a multi-compound array of bioactive agents. Rather than acting in isolation, these metabolites likely contribute through overlapping and synergistic mechanisms, conferring on this plant its wide range of pharmacological properties.

## 4. Traditional Uses of *M. indica* in Parasitic Diseases

For centuries, *M. indica* has been an integral part of traditional medicine in Africa, Asia, and South America. The plant is widely valued for treating a variety of ailments and several of its traditional indications are closely associated with parasitic infections (Table 2) [20,25].

In African ethnomedicine, *M. indica* was popularly used as an antidiarrheal medicinal plant, even in countries where it was considered an exotic plant, such as South Africa and Zimbabwe. Diarrheal and dysentery symptoms are often associated with infections caused by different types of intestinal parasites. Showing us that even when the plant was not specifically designated for parasitic infections, the spectrum of symptoms it was prescribed for, were commonly associated with such conditions [55]. Furthermore, its bark was used in Senegal, Ivory Coast, Nigeria and Congo, as medicine for dysentery, due to its astringent properties [54]. In India, the use of this plant, particularly of its seed kernel, as an antidiarrheal is also common. Furthermore, it was commonly administered with honey to treat helminthic infections and expel intestinal parasites. These broad traditional applications have sparked curiosity and encouraged early-stage studies on the phytochemical properties of this plant [56].

*M. indica* was also traditionally used for malaria in Africa, more specifically, in Cameroon. In a survey conducted in 2011 in the capital city, Maroua, about the use of medicinal plants against malaria, 58% of respondents reported the use of *M. indica* leaves and barks for this purpose. Traditional healers often combined mango bark extracts with other antipyretic plants in formulations and used them as an infusion to be taken as a drink, every six hours [57]. Similarly, a survey from Eastern Uganda, reported the same use of this species for the treatment of malaria and its symptoms, revealing a strong focus on using *M. indica* as an antipyretic [58]. These uses align with the plant’s later documented antiplasmodial activity, suggesting that traditional practice anticipated pharmacological validation [62].

Mango was also used as a source of topical treatments. Its gum was applied to the skin to soothe cracked skin and for scabies. Moreover, it was commonly used to cure ulcerated skin [59,60]. Although it may not be immediately associated with parasitic infections, the topical application of *M. indica* can be linked to such ailments. In the case of leishmaniases, the most common form of infection is cutaneous, causing skin ulcers usually on exposed parts of the body [63]. Thus, it is plausible to state that *M. indica*, although not directly, has been used as a natural product to soothe leishmaniasis symptoms.

Furthermore, *M. indica* has been reported as a plant of interest to treat ocular diseases [61]. The use of mango to treat eye infections or lesions, based on its ethnopharmaceuticals properties could, similarly to the case of leishmaniasis, have an indirect link to the treatment of parasitic eye infections, such as *Acanthamoeba* keratitis. Emphasizing another indirect use of mango in the treatment of parasitic diseases and its symptoms.

Such practices suggest traditional awareness of mango as a potential remedy for symptoms often associated with parasitic infections. Thus, this ethnopharmacological background provides an important rationale for investigating its antiparasitic potential in modern pharmacology.

## 5. Antiprotozoal Activity of *M. indica*

### 5.1. Malaria (Plasmodium spp.)

Malaria remains one of the deadliest parasitic diseases in the world, caused predominantly by *Plasmodium falciparum* and *P. vivax*, and transmitted to humans through the bite of a female *Anopheles* mosquito. Current treatment strategies rely primarily on artemisinin-based combination therapies (ACTs), whose long-term efficacy is increasingly threatened by the emergence of drug-resistant strains [64]. In this context, traditional medicine has inspired the search for novel phytochemicals with antimalarial potential, leading to the identification of several promising plant species [65]. Among them, *M. indica* has received particular attention, given its widespread ethnomedicinal use in the management of malaria and febrile illnesses (Table 2). In fact, among the endoparasitic diseases investigated to date, malaria is the one for which mango and its phytoconstituents have been most extensively studied [66].

In vitro studies have demonstrated that extracts derived from different parts of *M. indica* possess notable antiplasmodial properties. For example, a study by Cudjoe et al. reported that aqueous extracts of *M. indica* leaves completely inhibited *P. falciparum* growth at a concentration of 100 µg/mL [67]. Similarly, in a study by Koffi et al. compared aqueous, methanolic, and ethanolic bark extracts, showing that the ethanolic extract exhibited the strongest activity, with an IC_50_ of 19.08 µg/mL against a chloroquine-resistant strain [68]. Beyond crude extracts, mangiferin—one of the plant’s principal bioactive compounds—has also been investigated. A study by Maenpeun et al. demonstrated, through in vitro and in silico approaches, that mangiferin isolated from mango leaves could inhibit *P. falciparum* serine hydroxymethyltransferase (SHMT). SHMT plays an essential role in folate metabolism, catalyzing the reversible interconversion of serine and glycine, which is crucial for the synthesis of thymidylate and purines in Plasmodium. Inhibiting SHMT disrupts nucleotide biosynthesis and impairs parasite replication. Mangiferin’s reported binding affinity to SHMT suggests that it may interfere with this metabolic bottleneck, making this pathway a promising drug target [69].

Another proposed mechanism involves interference with the heme detoxification pathway, a well-established target of several existing antimalarial drugs. During infection, Plasmodium parasites degrade host hemoglobin, releasing free heme, which is toxic. To survive, the parasite detoxifies heme by polymerizing it into hemozoin (malaria pigment) [70]. Gupta et al. showed that 3-hydroxy-11-keto-β-boswellic acid, a terpene isolated from *Boswellia serrata*, inhibited this pathway [71]. As *M. indica* also contains diverse terpenoids, this raises the possibility that similar compounds may contribute to its antiplasmodial effects and warrants further exploration.

In vivo investigations have corroborated these findings, demonstrating that *M. indica* not only possesses antiplasmodial activity but also exerts hematoprotective and hepatoprotective effects. Plasmodium infection typically leads to erythrocyte lysis, anemia, and impaired oxygen transport, while also causing significant hepatocellular damage due to parasite sequestration and metabolic stress [65,72]. Moreover, recent in vivo studies reported that mango extracts were able to eliminate *P. berghei* schizonts in mice with 100% efficacy, while simultaneously mitigating biochemical alterations and restoring hepatocyte architecture [62,73].

Despite such encouraging findings, the precise molecular mechanisms underlying the antiplasmodial activity of *M. indica* remain incompletely understood. While inhibition of SHMT and interference with the heme detoxification pathway have been proposed, most evidence derives from in vitro assays and in silico predictions, with limited validation in animal models. Moreover, the individual contributions of different phytochemical classes present in mango—beyond mangiferin—are poorly defined.

### 5.2. Leishmaniasis (Leishmania spp.)

Leishmaniasis, caused by various species of the genus *Leishmania*, remains a significant therapeutic challenge due to the limited availability of effective drugs and the frequent occurrence of toxicity and resistance [74]. The ethnomedicinal use of *M. indica* bark preparations for the treatment of skin lesions and ulcers (Table 2) has prompted experimental investigations into its anti-leishmanial properties, although research in this area remains comparatively scarce relative to malaria.

In vitro studies have provided preliminary evidence supporting the activity of *M. indica* extracts against Leishmania. Petroleum ether, chloroform, and methanolic leaf extracts were shown to inhibit *L. donovani* promastigotes, with the methanolic extract exhibiting the strongest effect, reaching an IC_50_ of 2.74 µg/mL [75]. Essential oils from *M. indica* varieties Rosa and Espada have also been assessed against *L. amazonensis* promastigotes. The essential oil of *M. indica* var. Rosa—dominated by β-pinene and terpinolene—displayed an IC_50_ of 39.1 µg/mL, while that of var. Espada, primarily composed of terpinolene, demonstrated greater potency with an IC_50_ of 23.0 µg/mL [76]. Although mechanistic analyses were not conducted, it is noteworthy that the principal constituents of both oils are terpenes, a class of compounds with recognized immunomodulatory activities (Table 1). This highlights terpenoids as potential contributors to the anti-Leishmania activity of *M. indica*, as it has been demonstrated for other plant terpenoids that were able to induce reactive oxygen species formation and induce mitochondrial damage to the parasites [77,78].

Further supporting evidence comes from the work of García et al., who screened 46 Cuban plant species against *L. amazonensis*. Remarkably, only four—including *M. indica*—exhibited selective activity against promastigotes over macrophages, indicating a favorable therapeutic window and reduced risk of host cytotoxicity. Subsequent testing against intracellular amastigotes confirmed anti-leishmanial activity, with an IC_50_ of 60.1 µg/mL [79].

To date, mechanistic insights into the action of *M. indica* phytochemicals against Leishmania are lacking. Nonetheless, one of the most critical biological processes in *Leishmania* spp. is the polyamine biosynthetic pathway. This pathway is central to parasite proliferation and differentiation. [80] Enzymes such as ornithine decarboxylase and S-adenosylmethionine decarboxylase regulate the synthesis of spermidine and putrescine, which stabilize DNA and scavenge reactive oxygen species. [81,82,83] Plant-derived polyphenols like mangiferin and flavonoids may inhibit these enzymes directly or through oxidative stress modulation, thereby disrupting parasite redox balance and survival.

### 5.3. Trypanosomiasis (Trypanosoma spp.)

African trypanosomiasis (sleeping sickness), caused by *Trypanosoma brucei*, and American trypanosomiasis (Chagas disease), caused by *T. cruzi*, are life-threatening parasitic infections for which current therapeutic options remain inadequate. For Chagas disease, only two drugs are available, both of which are associated with limitations in efficacy and safety [84,85].

Evidence concerning the activity of *M. indica* or its phytochemicals against *Trypanosoma* spp. is extremely limited, with only two studies reported to date. The first, conducted by Awa-Imaga, evaluated the effect of aqueous leaf extract in *T. congolense*-infected rats. Treatment at the onset of infection reduced mortality compared to controls, an effect hypothesized to be linked to enhanced immune responses, as reflected by elevated plasma protein levels [86]. The second study, by Ohashi et al., screened 72 Ghanaian medicinal plants for antitrypanosomal activity and identified *M. indica* stem bark extract as active against *T. brucei*, with an IC_50_ of 77.37 µg/mL [87]. While preliminary, these findings suggest that mango-derived phytoconstituents could serve as starting points for the development of antitrypanosomal agents, though systematic validation is urgently needed.

To date, no mechanistic studies have explored how *M. indica* compounds might act against trypanosomiasis, underscoring a significant research gap. Target-based approaches could guide future investigations, focusing on essential parasite processes. One promising target is trypanothione reductase, a key enzyme in maintaining redox homeostasis, protecting the parasite against oxidative stress. This enzyme is parasite-specific making it widely recognized as critical for parasite survival [88]. In addition, as previously discussed for *Leishmania* spp., the polyamine biosynthesis pathway is indispensable for Trypanosoma viability. Inhibition of ornithine decarboxylase, a rate-limiting enzyme in polyamine synthesis, has already been highlighted as a potential therapeutic strategy [89]. Exploring whether *M. indica* phytochemicals can interfere with these pathways could provide important insights and open new avenues for drug discovery against trypanosomiasis.

### 5.4. Toxoplasmosis (Toxoplasma gondii)

*Toxoplasma gondii* is a globally prevalent protozoan parasite that can infect nearly all warm-blooded animals, including humans. In immunocompromised individuals, latent infections can reactivate, leading to severe clinical manifestations such as encephalitis, pneumonia, and disseminated systemic disease. Congenital transmission represents another major concern, as maternal infection during pregnancy can result in miscarriage, stillbirth, or lifelong neurological and ocular impairments in newborns [90].

To date, no studies have reported activity of *M. indica* or its phytochemicals against *T. gondii*. Nevertheless, several medicinal plants have demonstrated anti-toxoplasma properties, as highlighted in comprehensive reviews [91,92], reinforcing the promise of medicinal plants, such as mango, as potential sources of adjunct or alternative therapies for toxoplasmosis.

Beyond crude extracts, specific phytochemicals have also shown activity against *T. gondii*. For instance, β-amyrin—a triterpene belonging to a class of compounds present in *M. indica*—was found to significantly inhibit intracellular parasite proliferation, with an IC_50_ of 4.75 μg/mL [93]. Additionally, studies on non-plant natural products have expanded mechanistic insights relevant to future phytochemical investigations. The medicinal mushroom *Trametes versicolor* exhibited anti-toxoplasma activity with an IC_50_ of 5.98 μg/mL, linked to increased reactive oxygen species production and disruption of mitochondrial membrane potential [94].

Such findings highlight potential cellular targets—including oxidative stress pathways and mitochondrial integrity—that may also be relevant to mango-derived triterpenes and other phytoconstituents.

### 5.5. Amoebiasis and Acanthamoebiasis

Amoebiasis, caused by the protozoan *Entamoeba histolytica*, is a major cause of diarrheal disease worldwide and can lead to severe complications such as amoebic liver abscesses [95]. In addition, infections caused by free-living amoebae—including *Acanthamoeba* spp., *Naegleria fowleri*, and *Balamuthia mandrillaris*—are emerging as important causes of keratitis, encephalitis, and systemic disease, particularly in immunocompromised individuals [96]. These pathogens are environmentally widespread and frequently present diagnostic challenges due to their nonspecific clinical manifestations. Available therapies are limited, often associated with toxicity, and resistance has been increasingly documented in endemic regions. Collectively, these amoebic infections represent a neglected area of parasitology, highlighting the urgent need for improved diagnostic tools, surveillance, and novel therapeutic strategies [97,98].

The traditional use of *M. indica* bark and seed preparations for dysentery and other intestinal disorders (Table 2) suggests potential anti-*Entamoeba* activity, although no experimental studies have yet evaluated this hypothesis in vitro or in vivo. Insights can, however, be drawn from research on other medicinal plants. Extracts of *Tamarindus indica* and *Camellia sinensis* have shown activity against *E. histolytica*, with proposed mechanisms involving disruption of parasite cell membranes and inhibition of cyst formation [99]. By analogy, *M. indica* may share similar membrane-disruptive properties, warranting further investigation.

Research on free-living amoebae is even scarcer. Notably, the ethnomedicinal use of *M. indica* leaf infusions as eye washes for ocular infections has been associated with the management of keratitis-like symptoms, raising the possibility of activity against pathogens such as *Acanthamoeba* (Table 2). When considered alongside the beforementioned evidence of membrane-targeting effects in other plants, this traditional practice supports the hypothesis that mango-derived compounds may interfere with amoebic membrane integrity.

## 6. Anthelmintic Activity

The anthelmintic potential of *M. indica* has long been recognized in traditional medicine, particularly in the treatment of symptoms associated with helminthic infestations (Table 2). Helminthic diseases remain a major public health concern in low-resource settings, with nematodes, cestodes, and trematodes accounting for the majority of infections [100]. This ethnomedicinal background has stimulated scientific efforts to validate the efficacy of *M. indica* through standardized in vitro and in vivo assays.

De Macêdo et al. evaluated aqueous leaf extracts of *M. indica* against the nematode *Haemonchus contortus* using both in vitro and in vivo models. The extract, rich in tannins, flavones, and flavonoids, inhibited 81.6% of egg hatching at 30 mg/mL and significantly reduced larval development at 7.8 mg/g. In vivo, the extract produced a moderate reduction in fecal egg count, with an efficacy of 22.7% [101]. Similarly, Nery et al. investigated aqueous fruit extracts of *M. indica* against *Haemonchus* spp., reporting 100% inhibition of larval development at 100 mg/mL [102], a finding later corroborated by El-Sherbini and Osman [103].

Studies on earthworms (*Pheretima posthuma*) have further confirmed the anthelmintic potential of *M. indica*. Chattopadhyay et al., compared chloroform, ethanolic, and aqueous bark extracts, reporting that the ethanolic extract induced paralysis within 12.29 min at 80 mg/mL, faster than the other extracts but slower than the positive control (2.54 min) [104]. Extraction methodology was also addressed by Ruby et al., who showed that ethanolic extraction yielded a broader spectrum of phytochemicals compared to petroleum ether extraction, which produced only steroids. Their formulation with 120 g of ethanolic extract induced paralysis within 2.36–4.73 min, a range comparable to the positive control (2.1–3 min) [105].

The activity of isolated compounds has also been explored. García et al., demonstrated that mangiferin, administered at 50 mg/kg in a murine model of *Trichinella spiralis* infection, significantly reduced encysted larvae and adult worm burdens [106]. Interestingly, isolated mangiferin was more effective than crude aqueous extracts, underscoring the importance of identifying and characterizing specific active constituents.

Collectively, these studies highlight notable differences in efficacy depending on extraction methods and phytochemical composition. Ethanolic extracts appear particularly promising, as they yield a wider array of active compounds and demonstrate stronger activity in biological assays. Moreover, the superior efficacy of mangiferin compared to crude extracts suggests that targeted isolation and purification of bioactive compounds may enhance therapeutic outcomes.

Although the mechanisms underlying the anthelmintic effects of *M. indica* have not been elucidated, several plausible pathways can be inferred based on its phytochemical profile and on mechanisms established for other plant-derived anthelmintics. Tannins, which are abundant in the leaves and bark, are known to form complexes with cuticular glycoproteins and enzymes, leading to impaired nutrient absorption, cuticle disruption, and paralysis [107].

## 7. Main Findings, Research Gaps and Future Perspectives

The available evidence indicates that *M. indica* exhibits notable antiparasitic potential across both protozoa and helminth groups, though the strength and depth of data vary considerably. Among protozoa, the most consistent findings have been reported against *Plasmodium* spp., where both crude extracts and the isolated compound mangiferin demonstrated measurable activity. Several studies reported IC_50_ values in the low micromolar range, suggesting a degree of potency that is biologically relevant (Table 3). Evidence against *Leishmania* spp. is also encouraging, with methanolic leaf extracts and even essential oils inducing inhibition at low concentrations (Table 3). For trypanosomiasis evidence was very limited with only two available studies reporting anti-parasitic activity, revealing lower potency than that observed for Plasmodium and Leishmania (Table 3). Regarding toxoplasmosis, amoebiasis and acanthamoebiasis, evidence is currently nonexistent. However, as discussed, it is plausible to bridge the knowledge obtained from studies with other plants and even mushrooms, and hypothesize that *M. indica* could exhibit the same properties, due to some similarities in chemical composition (Table 3).

In contrast, the evidence for helminth control is not only broader but also functionally diverse. Both in vitro egg hatch assays and in vivo burden reduction studies consistently show that bark and leaf extracts exert substantial anthelmintic effects. Moreover, the studies conducted until today, show that extraction methodology plays a role in the anthelmintic efficacy, with ethanolic extract appearing to be the most effective procedure. Additionally, anthelmintic studies have even addressed the activity of isolated mangiferin, revealing it to be even more potent than crude extracts (Table 4). Collectively, these findings suggest that helminths, particularly nematodes, may be more sensitive to *M. indica* than many protozoa, at least under experimental conditions.

Regarding the influence of plant part and extraction method, it is also worth noting that bark and leaf extracts emerging as the most consistently active across parasites, especially in helminth models and protozoa such as Leishmania, likely due to their high content of polyphenols. Seeds and fruits, while rich in bioactive compounds, remain comparatively underexplored in parasitology studies, leaving a knowledge gap regarding their potential contribution. However, isolated compounds such as mangiferin showed more reproducible activity profiles, which is an advantage for pharmacological standardization. Even though synergy among phytochemicals in crude extracts may enhance efficacy, this is yet to be investigated.

Despite these promising findings, current research on *M. indica* as an antiparasitic agent faces important limitations. As reported in this review, the overwhelming majority of studies are confined to in vitro assays or small-scale in vivo experiments in animal models. Human clinical trials are entirely absent, which precludes translation into therapeutic applications. Another important limitation lies in the lack of methodological consistency across studies. Considerable variability exists in extraction procedures—ranging from differences in plant part selection, solvent polarity, concentration, and extraction time to the absence of yield reporting—which can markedly affect the phytochemical composition and, consequently, the observed biological activity. Likewise, as discussed in this review, studies have employed diverse parasite species or strains, inoculum sizes, and assay conditions, making cross-comparison of results difficult. Reported outcomes are often heterogeneous, with some studies expressing activity as IC_50_ values, others as percentage inhibition or qualitative observations, limiting direct comparability. To enhance reproducibility and facilitate data integration, future investigations should prioritize standardized approaches, such as the use of authenticated plant material with deposited voucher specimens; clearly defined and reproducible extraction protocols with solvent ratios and yield percentages reported; and in vitro and in vivo assay procedures aligned with internationally recognized guidelines. Results should be expressed in consistent quantitative units (e.g., µg/mL or µM) and supported by appropriate positive and negative controls, replicates, and statistical validation. Establishing such methodological uniformity will be essential for accurately assessing the antiparasitic potential of *M. indica* and for enabling future comparative or meta-analytical studies. Another weakness is the scarcity of mechanistic studies; while some work points to membrane disruption, oxidative stress induction, or interference with parasite energy metabolism, these hypotheses remain insufficiently validated. While the ethnopharmacological use of *M. indica* as an antiparasitic has been addressed, only a handful of studies attempt to systematically connect traditional knowledge with experimental pharmacology.

Together, the strongest evidence here presented supports the role of *M. indica* in the control of helminth infections, while antiprotozoal activity is more variable and requires further mechanistic and translational studies. Future research should prioritize standardized extract preparation, head-to-head comparisons of plant parts and solvents, mechanistic validation, and eventual movement toward controlled clinical studies. Only through this progression can the true potential of *M. indica* as a source of antiparasitic therapies be critically and reliably assessed.

Furthermore, future research should explore the advances in nanotechnology that offer a promising opportunity to enhance the therapeutic profile of *M. indica*-derived compounds. Bioactive molecules such as mangiferin often suffer from poor solubility and bioavailability, which may limit their systemic activity. Encapsulation into nanoemulsions, liposomes, or polymeric nanoparticles has already been shown in other plant-derived compounds to improve stability, targeted delivery, and cellular uptake [108,109,110]. Similar approaches could be applied to *M. indica*, particularly for ocular infections such as acanthamoebiasis or systemic protozoan diseases like leishmaniasis, where efficient delivery to specific tissues remains a major challenge.

Another underexplored aspect is the potential for synergism between *M. indica* extracts and existing antiparasitic drugs. In malaria, for instance, combining mangiferin or polyphenol-rich extracts with artemisinin-based therapies might improve efficacy while delaying the onset of drug resistance. Similar opportunities exist for leishmaniasis and trypanosomiasis, where polytherapy is often necessary to achieve effective parasite clearance. Testing combinations in vitro and in vivo could therefore provide valuable insights into the role of *M. indica* as an adjuvant therapy rather than a standalone agent [111,112].

Finally, in vivo toxicological and pharmacokinetic studies remain a critical priority. Despite its long history of traditional use, safety cannot be assumed at therapeutic doses or when concentrated extracts are administered systemically. Most studies have focused exclusively on in vitro assays or short-term in vivo experiments, without systematic assessment of acute or chronic toxicity, genotoxicity, or organ-specific effects. Similarly, key pharmacokinetic parameters—such as absorption, distribution, metabolism, excretion and bioavailability—are largely unknown for mangiferin and other major constituents. Without such information, it is impossible to establish safe and effective dosing regimens or predict potential interactions with existing antiparasitic drugs. To bridge this gap, future research should incorporate standardized toxicological screening, including dose–response and repeated-dose studies in suitable animal models. Detailed pharmacokinetic profiling using LC–MS/MS or related analytical techniques would clarify systemic exposure, metabolic stability, and tissue distribution. Once these preclinical parameters are well characterized, controlled pilot trials could evaluate safety, tolerability, and preliminary efficacy in human volunteers or animal infection models. Establishing this preclinical-to-clinical pipeline will be essential to translate *M. indica* from ethnopharmacological promise to validate therapeutic application [113,114].

While *M. indica* is not yet ready for translation into clinical applications, the evidence presented in this review underscores its promise as a multipurpose antiparasitic agent. Its ethnopharmacological legacy, rich phytochemistry, and encouraging experimental results provide a strong rationale for continued investigation. With systematic validation, integration of advanced drug delivery systems, and rigorous safety assessment, *M. indica* could emerge as a valuable adjunct or alternative therapy for parasitic diseases, bridging the gap between traditional medicine and modern pharmacology.

## 8. Conclusions

Parasitic diseases remain among the most pressing global health challenges, disproportionately affecting populations in tropical and subtropical regions. The increasing emergence of drug resistance, combined with limited accessibility and high costs of existing therapies, underscores the need for novel, safe, and affordable treatments.

This review highlights the diverse antiparasitic potential of *M. indica*, a plant long valued in traditional medicine and increasingly recognized in modern pharmacological research. Extracts from different plant parts—particularly leaves and bark—along with the xanthone mangiferin and other phytoconstituents, have demonstrated activity against protozoa (Plasmodium, Leishmania, Trypanosoma) and helminths. The strongest and most consistent evidence exists for antiplasmodial and anthelmintic activities, with both crude extracts and purified compounds exhibiting biologically relevant effects. Although data for other protozoa remains absent, phytochemical similarities to plants with demonstrated activity suggest potential worth exploring.

*M. indica* represents an affordable, widely available, and sustainable resource for communities in endemic regions where access to conventional drugs remains limited. The strengths of current evidence lie in its ethnopharmacological foundation and the breadth of in vitro and in vivo assays demonstrating activity across diverse parasite groups. Isolated compounds such as mangiferin add translational value by providing reproducible pharmacological profiles and opportunities for standardization.

## Figures and Tables

**Table 1:** Major phytochemicals of *M. indica* and their pharmacological properties.

Compound/Class	Chemical Structure	Plant Part (Main Source)	General Pharmacological Properties	References
Mangiferin (xanthone)	Xanthone derivative	Leaves, bark	Antioxidant, anti-inflammatory, immunomodulatory	[43,44]
Phenolic acids (gallic, caffeic, chlorogenic, ellagic)	Carboxylic acid derivatives	Peel	Antioxidant, antimicrobial, enzyme inhibition	[45,46,47]
Flavonoids (quercetin, catechins, kaempferol, rutin)	Polyphenolic compounds	Leaves, peel, fruit	Anti-inflammatory, antioxidant, membrane stabilization, signaling modulation	[23,48,49,50]
Tannins (condensed, hydrolyzable)	Oligomeric flavonoids	Leaves, fruit, kernel, stem bark	Antimicrobial, anti-diarrheal, protein-binding, tissue-protective	[51,52]
Triterpenes & Sterols (lupeol, β-sitosterol, cycloartanes)	Steroid-like structures	Bark, seeds, kernels	Anti-inflammatory, immunomodulatory	[20]

**Table 2:** Traditional uses of *M. indica* in parasitic and related diseases.

Region	Plant Part Used	Preparation	Traditional Indication (Parasitic Link)	Reference
Senegal, Ivory Coast, Nigeria, Congo	Bark	Decoction/infusion	Dysentery, diarrhea (possible link: intestinal parasites)	[54]
South Africa, Zimbabwe	Bark, seeds	Powder, decoction	Antidiarrheal (possible link: intestinal parasites)	[55]
India	Seed kernel	Powder (with honey)	Helminthic infestations	[56]
Cameroon, Uganda	Leaves, bark	Infusion, decoction	Malaria, febrile illnesses (*Plasmodium* spp.)	[57,58]
India, Asia	Gum, bark	Topical	Ulcers, cracked skin, scabies (possible link: leishmaniasis)	[59,60]
Asia	Leaves (infusion), bark	Eye wash, poultice	Eye infections (possible link: *Acanthamoeba* keratitis)	[61]

**Table 3:** Reported antiparasitic activity of *M. indica* against protozoa.

Parasite/Disease	Experimental Model	Extract/Compound	Potency (IC_50_, MIC, Survival, etc.)	Proposed Mechanism(s)	Evidence Strength	References
*Plasmodium* spp. (Malaria)	*In vitro* (*P. falciparum*, chloroquine-resistant & sensitive strains)	Aqueous/ethanolic leaf & bark extracts	100% inhibition at 100 µg/mL (leaf aqueous); bark ethanolic IC_50_ = 19.08 µg/mL	Heme detoxification interference; ROS imbalance; mitochondrial disruption	Strong (multiple in vitro studies)	[67,68]
	*In vitro* (molecular target)	Mangiferin (leaf isolate)	Inhibition of serine hydroxymethyltransferase (SHMT)	Enzyme inhibition (SHMT blockade)	Moderate–Strong (in vitro & in silico evidence)	[69]
	*In vivo* (*P. berghei*-infected mice)	Crude extracts (various solvents)	100% schizont elimination; reduced parasitemia; hepatoprotective & haematoprotective effects	Enzyme inhibition (SHMT blockade)	Strong (animal validation)	[62,73]
*Leishmania* spp. (Leishmaniasis)	*In vitro* (*L. donovani* promastigotes)	Petroleum ether, chloroform, methanol leaf extracts	Methanol extract IC_50_ = 2.74 µg/mL	Not defined	Moderate	[75]
	*In vitro* (*L. amazonensis* promastigotes)	Essential oils (var. Rosa, Espada)	Rosa IC_50_ = 39.1 µg/mL; Espada IC_50_ = 23.0 µg/mL	Terpene-driven activity (β-pinene, terpinolene)	Moderate	[76]
	*In vitro* (*L. amazonensis* promastigotes & amastigotes)	Crude extract	Selective against promastigotes; IC_50_ = 60.1 µg/mL (amastigotes)	Possible polyamine biosynthesis inhibition (ornithine decarboxylase)	Moderate	[79]
*Trypanosoma* spp. (Trypanosomiasis)	*In vivo* (*T. congolense*-infected rats)	Aqueous leaf extract	Reduced mortality; higher plasma proteins; survival benefit	Hypothesized immune stimulation	Weak (single preliminary study)	[86]
	*In vitro* (*T. brucei*)	Stem bark crude extract	IC_50_ = 77.37 µg/mL	Possible enzyme inhibition (trypanothione reductase, polyamine pathway)	Weak (single screen)	[87]
*Toxoplasma gondii* (Toxoplasmosis)	No direct studies	No direct studies	No direct studies	Hypothetical activity via triterpenes (e.g., β-amyrin); mitochondrial/ROS disruption based on analogues	Very weak (no direct evidence)	[93,94]
*Entamoeba histolytica*/*Acanthamoeba* spp. (Amoebiasis & Acanthamoebiasis)	No direct studies	No direct studies	No direct studies	Hypothetical membrane disruption; inhibition of cyst formation	Very weak (no direct experimental evidence)	[61,99]

**Table 4:** Reported antiparasitic activity of *M. indica* against helminths.

Parasite/Model	Extract/Compound	Experimental System	Main Findings (IC_50_, % Inhibition, Worm Burden Reduction)	References
*Haemonchus contortus* (nematode)	Aqueous leaf extract (tannins, flavones, flavonoids)	*In vitro* egg hatch and larval development assays; *in vivo* infection model	81.6% egg hatch inhibition at 30 mg/mL; significant reduction in larval development at 7.8 mg/g; in vivo: moderate efficacy, fecal egg count reduction 22.7%	[101]
*Haemonchus* spp. (nematode)	Aqueous fruit extract	*In vitro* larval development assay	100% inhibition of larval development at 100 mg/mL	[102]
*Haemonchus* spp. (nematode)	Aqueous fruit extract	*In vitro* larval development assay	Confirmed 100% inhibition of larval development	[103]
*Pheretima posthuma* (earthworm model)	Bark extracts (chloroform, ethanolic, aqueous)	*In vitro* paralysis assay (pin-prick method)	Ethanolic extract induced paralysis in 12.29 min at 80 mg/mL (vs. 2.54 min positive control)	[104]
*Pheretima posthuma* (earthworm model)	Ethanolic bark extract formulation	*In vitro* paralysis assay	Paralysis achieved in 2.36–4.73 min, comparable to positive control (2.1–3 min); petroleum ether yielded only steroids, less active	[105]
*Trichinella spiralis* (nematode)	Isolated mangiferin vs. crude aqueous extract	*In vivo *mouse infection	Mangiferin (50 mg/kg) significantly reduced encysted larvae and adult worms; more effective than crude extract	[106]

## Data Availability

No new data were created or analyzed in this study. Data sharing is not applicable to this article.
